# Repeat HIV-testing is associated with an increase in behavioral risk among men who have sex with men: a cohort study

**DOI:** 10.1186/s12916-015-0458-5

**Published:** 2015-09-11

**Authors:** Martin Hoenigl, Christy M. Anderson, Nella Green, Sanjay R. Mehta, Davey M. Smith, Susan J. Little

**Affiliations:** AntiViral Research Center, Division of Infectious Diseases, Department of Medicine, University of California, San Diego, 200 West Arbor Drive #8208, San Diego, CA 92103 USA; Section of Infectious Diseases and Tropical Medicine, Department of Internal Medicine, Medical University of Graz, Graz, Austria; Division of Pulmonology, Department of Internal Medicine, Medical University of Graz, Graz, Austria; Veterans Affairs Healthcare System, San Diego, CA USA

**Keywords:** Acute and early HIV, MSM, Risk behavior, NAT screening, Repeat testing

## Abstract

**Background:**

The Center for Disease Control and Prevention recommends that high-risk groups, like sexually active men who have sex with men (MSM), receive HIV testing and counseling at least annually. The objective of this study was to investigate the relationship between voluntary repeat HIV testing and sexual risk behavior in MSM receiving rapid serologic and nucleic acid amplification testing.

**Methods:**

We performed a cohort study to analyze reported risk behavior among MSM receiving the “Early Test”, a community-based, confidential acute and early HIV infection screening program in San Diego, California, between April 2008 and July 2014. The study included 8,935 MSM receiving 17,333 “Early Tests”. A previously published risk behavior score for HIV acquisition in MSM (i.e. Menza score) was chosen as an outcome to assess associations between risk behaviors and number of repeated tests.

**Results:**

At baseline, repeat-testers (n = 3,202) reported more male partners and more condomless receptive anal intercourse (CRAI) when compared to single-testers (n = 5,405, all *P* <0.001). In 2,457 repeat testers there was a strong association observed between repeated HIV tests obtained and increased risk behavior, with number of male partners, CRAI with high risk persons, non-injection stimulant drug use, and sexually transmitted infections all increasing between the first and last test. There was also a linear increase of risk (i.e. high Menza scores) with number of tests up to the 17th test. In the multivariable mixed effects model, more HIV tests (OR = 1.18 for each doubling of the number of tests, *P* <0.001) and younger age (OR = 0.95 per 5-year increase, *P* = 0.006) had significant associations with high Menza scores.

**Conclusions:**

This study found that the highest risk individuals for acquiring HIV (e.g. candidates for antiretroviral pre-exposure prophylaxis) can be identified by their testing patterns. Future studies should delineate causation versus association to improve prevention messages delivered to repeat testers during HIV testing and counseling sessions.

## Background

Men who have sex with men (MSM) bear the greatest burden of HIV infection in California and the United States [1–5]. The Center for Disease Control and Prevention recommends that high-risk groups, like sexually active MSM, receive HIV testing and counseling at least annually [6]. To address this recommendation, community-based, confidential HIV screening programs that include screening for acute HIV (i.e. detect HIV antigen in antibody negative persons), like the “Early Test” in San Diego, California [7], have been implemented in many US metropolitan areas. Higher rates of repeat testing and counseling have been reported in persons with more behavioral risks for HIV acquisition [8–10], though most of these studies were conducted either in resource limited settings or more than 10 years ago (i.e. before the era of rapid HIV antibody or acute HIV infection screening) [11–13].

HIV testing and counseling typically includes an assessment of recent behavioral risks, pre-test counseling (in some settings), and rapid provision of HIV test results. Detection of HIV infection is often associated with at least transiently reduced risk behaviors and thus a decreased risk of HIV transmission [14, 15]. When test results are negative, however, the client could interpret this as positive reinforcement that ongoing risk behaviors are not sufficiently risky to result in HIV infection [16]. In other words, reported risk behaviors without significant consequences may foster greater risk in the future [17]. Thus, repeated negative HIV test results over time may provide an unintentionally reassuring message that may ultimately contribute to higher HIV-acquisition rates among repeat testers. In particular, acute infection screening (i.e. nucleic acid amplification testing) provides HIV status information related to very recent risk behaviors as well as greater certainty about a negative HIV test result [18]. We investigated the relationship between voluntary repeat HIV testing and sexual risk behavior in MSM receiving rapid serologic testing and nucleic-acid testing (NAT).

## Methods

In this retrospective analysis of a prospective cohort study, we analyzed risk behavior reported for the previous 12 months in individuals who enrolled in the “Early Test” HIV screening program between April 2008 and July 2014. The “Early Test” [19] is a community-based, voluntary, confidential acute and early HIV infection (AEH) screening program that provides point of care rapid HIV serologic testing followed by routine reflex to individual donation HIV NAT (NAT results are provided in a second visit or, if negative, also with automated voice mail or online) in all antibody-negative persons [7, 20, 21]. With the “Early Test” program approximately 4000 individuals per month are screened free of charge at five regular plus additional mobile testing sites (including sites targeting MSM at the Lesbian, Gay, Bisexual, Transgender Center and the Gay Men’s Health Clinic; the San Diego County Health Department; the AntiViral Research Center (AVRC); substance abuse treatment centers; and special community event venues) in San Diego, United States. Similar to other settings in the United States, MSM bear by far the greatest burden of HIV infection in San Diego, as reflected in the “Early Test”, where 72 % of tests overall, 85 % of repeat testing encounters, and 88 % of HIV diagnoses are made to MSM. Since the “Early Test” does not routinely keep track of individuals who repeatedly test over time, tests were considered linked to the same individual if the birth date and soundex of last-name and first-name matched.

**Fig. 1:**
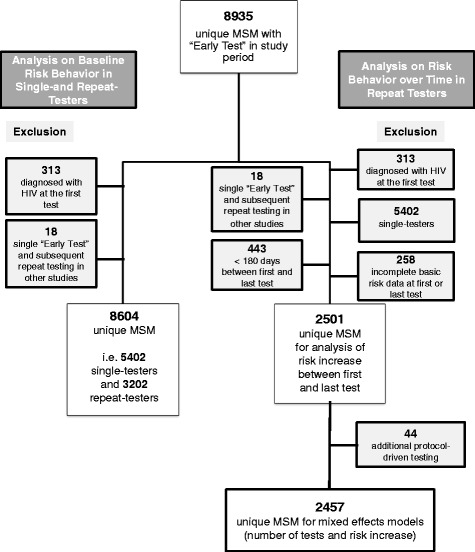
Tree of enrollment for analyses on baseline risk-taking behavior in single- and repeat-testers and risk behavior increase in repeat-testers (reasons/number of excluded individuals are displayed in grey shapes)

Pre-test counseling was provided at each Early Test visit throughout the study period and utilized a client-centered harm reduction model offering personalized risk reduction options. Risk behavior was collected by using a risk assessment form with 19 detailed survey questions (focusing primarily on sexual risk behavior, substance use, sexually transmitted infection (STI) diagnoses – all reported for the prior 12 months – and demographics). Survey questions were assessed and the form filled out by the testing staff before each HIV testing encounter, and data was later entered into the data system (always in duplicate to minimize data entry errors). After testing, clients were recommended to come back 6 months later for the next testing encounter. Males and female-to-male transsexual persons who reported sexual contact with one or more male partners during the previous 12 months were included in the analysis. We excluded (1) those who were diagnosed with HIV (irrespective of HIV stage) at the first test since these participants did not have the opportunity to become repeat testers, and (2) those with only one “Early Test” who had repeat tests required as part of their participation in another study (Fig. 1). AEH was characterized using previously published criteria for serologic and virologic test results [22]. We evaluated the predictive potential (for AEH versus HIV negatives) of a previously published risk behavior score for HIV acquisition in MSM repeat testers [23] as an outcome measure to assess changes in risk behavior. The shorter version of this score (i.e. the Menza score), included four variables: diagnosis or history of STI, use of methamphetamine or inhaled nitrites (i.e. poppers) in the prior 6 months, 10 or more male sex partners in the prior year, and unprotected anal intercourse with someone who is HIV positive in the prior year [23]. Two modifications were necessary for our study: (1) diagnosis or history of STI was modified to self-reported diagnosis of STI within the last 12 months, and (2) time period of methamphetamine or inhaled nitrite use was increased to last 12 months (as we did not have data available for last 6 months). Performance of the Menza score in our cohort was evaluated using receiver operating characteristics (ROC) analysis and area under the curve (AUC) value, including 95 % confidence interval (CI) displayed.

Our main analyses focused on: (1) baseline risk behavior in single- and repeat- testers, and (2) risk behavior over time in repeat testers.

### Baseline risk behavior in single and repeat testers

For the analysis of baseline risk behavior, we evaluated the differences in risk behavior reported at the initial HIV screening between single testers and repeat testers. To evaluate the difference between single and repeat testers, we compared the baseline risk-taking behaviors in the 12 months prior to testing using *χ*^2^ and Mann Whitney *U* test. To identify baseline characteristics that were associated with repeat testing, we used Cox proportional-hazards regression, modeling the time from initial test to (1) second test for repeat testers or (2) end of follow-up period for single testers. Univariate analyses were performed, and variables with *P* <0.20 were included in the multivariable model. Variables in the final model were selected with a forward stepwise procedure. Hazard ratios (HR) including 95 % CI were displayed.

### Risk behavior at first and last test in repeat testers

For the comparison of risk behavior reported at the first and last test in repeat testers, those with incomplete basic risk data at their first or last test (i.e. number of male partners, STI and drug use), and those who had their first and last test less than 180 days apart were additionally excluded (as analysis focused on risk behavior reported for the prior 12 months, Fig. 1). Analyses of changes in risk behaviors between the first and the most recent “Early Test” used the McNemar test and Wilcoxon signed rank test.

### Risk behavior and number of tests in repeat testers

Further, individuals with repeat “Early Test” visits who received additional protocol-driven interval tests as part of co-enrollment in another study (n = 44) were excluded from the analyses to evaluate risk behavior over time in repeat testers (Fig. 1). Analyses of changes in risk behaviors between the first and the most recent “Early Test” was performed separately for those with 2 to 3, 4 to 5, and 6 or more tests as described above.

Risk behavior at each Early Test visit was further characterized with the previously published Menza score [23]. Since scores clustered around awarded points based on four specific risk behaviors (i.e. weighted scores for risk behavior vary between 1 and 11), and therefore did not follow a discrete-count distribution, we dichotomized scores based on the median: high risk (above the mean of individuals’ median scores) or low risk (below the mean). At each time point, the cumulative number of HIV tests was used as the primary predictor and the effect of the number of tests was investigated while controlling for follow-up time and age. Percentage of high (i.e. above the mean) HIV behavioral risk scores by number of HIV tests was calculated for repeat testers using local regression (Loess), a smoothing technique that allows one to see a pattern without assuming a particular distribution. Visual and Loess fitting suggested that there was a linear relationship between the number of HIV tests and the risk-behavior score up to approximately the 17th test. As subsequent tests (from 18th test onward) did not correspond to the same increase in higher scores, numbers of tests were log-transformed to achieve a linear relationship between number of tests and the logit of high-risk scores.

Univariable and multivariable mixed-effects logistic regression models were performed for number of tests, time since first HIV test and age, and odds ratios (OR) including 95 % CI were displayed. In addition, associations between the number of tests (log_2_) and individual known risk behaviors for HIV that were not included in the Menza score were assessed by univariable mixed-effects logistic regression models.

For statistical analysis SPSS 21 (SPSS Inc., Chicago, IL, USA) and SAS 9.4 (SAS Institute, Cary, NC) were used.

The UCSD Human Research Protections Program approved the study protocol, consent, and all study related procedures. All study participants provided voluntary, written informed consent before any study procedures were undertaken.

## Results

A large population (n = 14,612 unique clients) underwent HIV screening using the “Early Test” between April 2008 and July 2014, including 8,935 (61 %) unique MSM (with 17,333 voluntary HIV tests). Notably, 10 % (n = 892) of MSM participants reported sex with women in the past year. Overall, 419 of 8,935 MSM (4.69 %) were newly diagnosed with HIV infection, 219 (2.45 %) with chronic HIV infection, and 200 (2.24 %) with AEH (125/200 (63 %) at their first “Early Test”, 75/200 (37 %) at a repeated visit). Incidence rate among MSM repeat testers was 1.783 per 100 person years. The Menza score [23] for HIV infection among MSM was significantly higher in those with AEH than in those without HIV (median 3 points, interquartile range (IQR) 0–11 versus median 0 point, IQR 0–4, *P* <0.001) and displayed a moderate AUC value of 0.636 (95 % CI, 0.594–0.677) for prediction of AEH.

### Baseline risk behavior in single and repeat testers

A comparison of baseline risk behaviors in the 12 months prior to testing and demographics between single and repeat testers is shown in Table 1. A total of 5,402 (63 %) single and 3,202 (37 %) repeat testers (Fig. 1) were included in the analysis. Repeat testers had up to 30 HIV tests during a mean period of follow-up of 104 weeks (standard deviation 84 weeks). Approximately 50 % of repeat testers had 2 or 3 tests and another 25 % (n = 814) had 5 or more tests. At baseline, repeat testers reported significantly more male partners and more condomless receptive anal intercourse (CRAI), and fewer reported sexual contacts with females when compared to single testers (all *P* <0.001).

**Table 1:** Comparison demographics and risk behavior at baseline. Reported by individuals with a single HIV test during the study period versus those with repeated HIV tests (those with two or more tests, and the subpopulation with five or more tests)

Risk factor/risk behavior (within 12 months prior to test) and demographics	Single testers ^a^	Repeat testers (2 or more tests) ^a^	*P* value ^b^	Repeat tester (5 or more tests) ^a^	*P* value ^b^
N	5402	3202		814	
Number of male partners (median, IQR)	5 (3–10)	6 (3–12)	<0.001	6 (4–14)	<0.001
10 or more male partners	1624/5397 (30.1 %)	1120/3197 (35.0 %)	<0.001	319 (39.2 %)	<0.001
Intercourse with females also	594/5389 (11.0 %)	277/3192 (8.7 %)	<0.001	64/813 (7.9 %)	0.007
CIAI with male	3188/5347 (59.6 %)	1974/3170 (62.3 %)	0.016	491/804 (61.1 %)	n.s.
CRAI	2611/5343 (48.9 %)	1664/3167 (52.5 %)	0.001	434/802 (54.1 %)	0.003
CRAI and 5 or more male partners	1544/5342 (28.9 %)	1102/3165 (34.8 %)	<0.001	306/802 (38.2 %)	<0.001
CRAI and 10 or more male partners	892/5342 (16.7 %)	632/3165 (20.0 %)	<0.001	181/802 (22.6 %)	<0.001
CRAI with HIV positive	214/5097 (4.2 %)	121/2947 (4.1 %)	n.s.	19/725 (2.6 %)	0.043
CRAI with PWID	56/5281 (1.1 %)	29/3129 (0.9 %)	n.s.	4/790 (0.5 %)	n.s.
CRAI with sex worker	21/5147 (0.4 %)	9/3052 (0.3 %)	n.s.	1/764 (0.1 %)	n.s.
Worked as sex worker	84/3799 (2.2 %)	45/2004 (2.3 %)	n.s.	8/424 (1.9 %)	n.s.
Syphilis ^c^	101 (1.9 %)	59 (1.8 %)	n.s.	23 (2.8 %)	n.s.
Gonorrhea ^c^	274 (5.1 %)	193 (6.0 %)	n.s.	47 (5.8 %)	n.s.
Chlamydia ^c^	214 (4.0 %)	129 (4.0 %)	n.s.	48 (5.9 %)	0.01
Any STI ^c^	596 (11.0 %)	402 (12.6 %)	0.033	126 (15.5 %)	<0.001
Methamphetamine, not injected	363 (6.7 %)	189 (5.9 %)	n.s.	44 (5.4 %)	n.s.
Non-injection stimulant drug use (i.e. methamphetamine, cocaine, poppers, GHB, ketamine, XTC)	1316 (24.4 %)	765 (23.9 %)	n.s.	176 (21.6 %)	n.s.
IDU	77 (1.4 %)	37 (1.2 %)	n.s.	5 (0.6 %)	n.s.
IDU with shared needles	35/5378 (0.7 %)	7/3180 (0.2 %)	0.006	0	0.021
Demographic data					
Age (years; median, IQR)	33 (26–43)	32 (26–42)	0.011	32 (26–41)	n.s.
Male	5402 (100 %)	3202 (100 %)	n.s.	814 (100 %)	
Hispanic origin	1434/5249 (27.3 %)	850/3105 (27.4 %)	n.s.	203/785 (25.9 %)	n.s.
Race	–	–	<0.001	–	<0.001
Caucasian	3513/5066 (69.3 %)	2112/3021 (69.9 %)	n.s.	547/783 (69.9 %)	n.s.
African-American	317/5066 (6.3 %)	143/3021 (4.7 %)	0.005	39/783 (5.0 %)	n.s.
Asian	398/5066 (7.9 %)	195/3021 (6.5 %)	0.022	52/783 (6.6 %)	n.s.
Pacific Islander	142/5066 (2.8 %)	53/3021 (1.8 %)	0.004	11/783 (1.4 %)	0.030
Native American	37/5066 (0.7 %)	15/3021 (0.5 %)	n.s.	1/783 (0.1 %)	n.s.
Other	659/5066 (13.0 %)	503/3021 (16.7 %)	<0.001	133/783 (17.0 %)	0.002

CIAI, Condomless insertive anal intercourse; CRAI, Condomless receptive anal intercourse; GHB, Gamma hydroxybutyrate; IDU, Injection drug use; IQR, Inter-quartile range; MSM, Men who have sex with men; n.s., Not significant; PWID, Person who injects drugs; STI, Sexually transmitted infection; XTC, Ecstasy

^a^ Data available from all individuals if denominator not depicted

^b^ Calculated using *χ* ^2^ or Mann–Whitney *U*-test

^c^ All self-reported diagnosis within the last 12 months

In the multivariable analysis, CRAI (HR, 1.089; 95 % CI, 1.01–1.173; *P* = 0.026), 10 or more male partners (HR, 1.189; 95 % CI, 1.1–1.285; *P* <0.001), and not having sexual contact with females also (HR, 0.824; 95 % CI, 0.725–0.936; *P* = 0.003) were associated with repeat testing (also included in the model: injection drug use, CRAI with a HIV positive male, and STI).

### Risk behavior at first and last test in repeat testers

Risk behaviors reported at the first and last test are summarized in Table 2 for 2,501 repeat testers (Fig. 1) with more than 180 days between their first and last test (median time between first and last test 714 days, IQR, 396–1191). Demographic characteristics of the evaluable 2,501 repeat testers reflected those observed for repeat testers in Table 1. Using Wilcoxon’s signed rank sum test, repeat testers reported significantly more male partners (*P* = 0.001) and fewer female partners (*P* <0.001) at the last test when compared to the first. Further, the proportions of individuals reporting 10 or more male partners alone or in combination with CRAI, CRAI with an HIV-positive male, CRAI with a person who injects drugs (PWID), gonorrhea, and non-injection stimulant drug use (i.e. methamphetamine, cocaine, poppers, gamma hydroxybutyrate, ketamine, ecstasy) were significantly higher at the last test when compared to the first (Table 2).

**Table 2:** Risk behavior at the first and last test in 2,501 individuals undergoing repeat HIV testing ^a^

Risk factor/risk behavior (within 12 months prior to test)	First test		Last test		
	Individuals reporting risk	Percentage	Individuals reporting risk	Percentage	*P* value using McNemar test
10 or more male partners	871/2501	34.83 %	970/2501	38.78 %	<0.001
CRAI	1308/2474	52.87 %	1323/2490	53.13 %	n.s.
CRAI and 5 or more male partners	859/2483	34.60 %	915/2495	36.67 %	0.028
CRAI and 10 or more male partners	488/2483	19.65 %	580/2495	23.25 %	<0.001
CRAI with HIV positive male	76/2288	3.32 %	161/2422	6.65 %	<0.001
CRAI with sex worker	7/2436	0.29 %	12/2467	0.49 %	n.s.
CRAI with PWID	14/2437	0.57 %	40/2464	1.62 %	0.002
Gonorrhea ^b^	141/2501	5.64 %	189/2501	7.56 %	0.004
Syphilis ^b^	50/2501	2.00 %	47/2501	1.88 %	n.s.
Any STI ^b^	317/2501	12.67 %	348/2501	13.91 %	n.s.
IDU with shared needles	9/2495	0.36 %	19/2501	0.76 %	n.s.
IDU	26/2501	1.04 %	28/2501	1.12 %	ns
Non-injection stimulant drug use (i.e. methamphetamine, cocaine, poppers, GHB, ketamine, XTC)	562/2501	22.47 %	759/2501	30.35 %	<0.001

CRAI, Condomless receptive anal intercourse; GHB, Gamma hydroxybutyrate; IQR, Interquartile range; IDU, Injection drug use; n.s., Not significant; PWID, Person who injects drugs; STI, Sexually transmitted infection; XTC, Ecstasy

^a^ Median time between first and last test 714 days (IQR, 396–1191); individuals with <180 days between tests were excluded

^b^ All self-reported diagnosis within the last 12 months

### Risk behavior and number of tests in repeat testers

We also investigated risk increase between first and last test by number of tests by dividing the study cohort in three groups: those with 2 to 3 HIV tests (n = 1,384), those with 4 to 5 tests (n = 565), and those with 6 or more tests (n = 508). There was a positive association between number of tests and CRAI with an HIV-positive male, CRAI with a PWID, and non-injection stimulant drug use (Table 3).

**Table 3:** Risk behavior reported at the first and last test in individuals undergoing repeat HIV testing: individuals with <180 days between tests were excluded

		2–3 HIV tests (n = 1384; 47 HIV+) median time first/last test 503 days (IQR, 317–845 days)				4–5 HIV tests (n = 565; 22 HIV+) median time first/last test 844 days (IQR, 567–1229 days)				6 or more tests (n = 508; 18 HIV+) median time first/last test 1333 days (IQR, 920–1705 days)			
Risk factor/risk behavior (within 12 months prior to test)	Prevalence of AEH per individuals that reported the risk ^a^	First Test	Last Test			First Test	Last Test			First Test	Last Test		
		Individuals reporting risk (n,%)	Individuals reporting risk (n, %)	Difference in percentage, relative (absolute)	*P* value ^b^	Individuals reporting risk (n, %)	Individuals reporting risk (n, %)	Difference in percentage, relative (absolute)	*P* value ^b^	Individuals reporting risk (n, %)	Individuals reporting risk (n, %)	Difference in percentage, relative (absolute)	*P* value ^b^
10 or more male partners	3.8 %	456/1384 (32.95 %)	504/1384 (36.42 %)	+10.53 % (+3.47 %)	0.011	193/565 (34.16 %)	224/565 (39.65 %)	+16.07 % (+5.49 %)	0.012	198/508 (38.98 %)	223/508 (43.90 %)	+12.62 % (+4.92 %)	0.054
CRAI and 10 or more male partners	5.5 %	255/1384 (18.43 %)	301/1384 (21.75 %)	+18.01 % (+3.32 %)	0.006	101/565 (17.88 %)	132/565 (23.36 %)	+30.65 % (+5.48 %)	0.004	116/508 (22.83 %)	130/508 (25.59 %)	+12.09 % (+2.76 %)	n.s.
CRAI with HIV positive male	7.8 %	46/1277 (3.60 %)	72/1335 (5.39 %)	+49.72 % (+1.79 %)	n.s.	18/526 (3.42 %)	32/551 (5.81 %)	+69.88 % (+2.39 %)	n.s.	8/446 (1.79 %)	52/495 (10.51 %)	+487 % (+8.72 %)	<0.001
CRAI with sex worker	7.7 %	5/1350 (0.37 %)	6/1364 (0.44 %)	+10.45 % (+0.07 %)	n.s.	1/555 (0.18 %)	2/561 (0.36 %)	+100 % (+0.18 %)	n.s.	1/489 (0.20 %)	4/502 (0.80 %)	+300 % (+0.60 %)	n.s.
CRAI with PWID	10.3 %	9/1350 (0.67 %)	17/1365 (1.25 %)	+86.57 % (+0.58 %)	n.s.	5/555 (0.90 %)	13/559 (2.33 %)	+159 % (+1.43 %)	n.s.	0/491 (0 %)	9/498 (1.81 %)	+∞ (+1.81 %)	0.008
Any self-reported STI	4.6 %	155/1384 (11.20 %)	177/1384 (12.79 %)	+14.20 % (+1.59 %)	n.s.	64/565 (11.33 %)	87/565 (15.40 %)	+35.92 % (+4.07 %)	0.040	91/508 (17.91 %)	77/508 (15.16 %)	−15.35 % (−2.75 %)	n.s.
IDU with shared needles	7.8 %	4/1384 (0.29 %)	9/1384 (0.65 %)	+124 % (+0.36 %)	n.s.	2/565 (0.35 %)	5/555 (0.90 %)	+157 % (+0.55 %)	n.s.	0/508 (0 %)	3/508 (0.59 %)	+∞ (+0.59 %)	n.s.
Methamphetamine, not injected	6.2 %	77/1384 (5.56 %)	88/1384 (6.36 %)	+14.39 % (+0.80 %)	n.s.	28/565 (4.96 %)	35/565 (6.19 %)	+24.79 % (+1.23 %)	n.s.	26/508 (5.11 %)	38/508 (7.48 %)	+46.38 % (+2.37 %)	0.090
Non-injection stimulant drug use ^c^	3.3 %	318/1384 (22.98 %)	411/1384 (29.70 %)	+29.24 % (+6.72 %)	<0.001	130/565 (23.01 %)	166/565 (29.38 %)	+27.58 % (+6.37 %)	0.002	96/508 (18.90 %)	163/508 (32.09 %)	+69.79 % (+13.19 %)	<0.001

AEH, Acute and early HIV infection; CRAI, Condomless receptive anal intercourse; IQR, Interquartile range; IDU, Injection drug use; n.s., Not significant; PWID, Person who injects drugs; STI, Sexually transmitted infection; ^a^ Among whole MSM study population after exclusion of those with newly diagnosed chronic HIV infection

^b^ Calculated using McNemara test; *P* value displayed when below 0.1

^c^ i.e. Methamphetamine, cocaine, poppers, Gamma hydroxybutyrate, ketamine, ecstasy

**Fig. 2:**
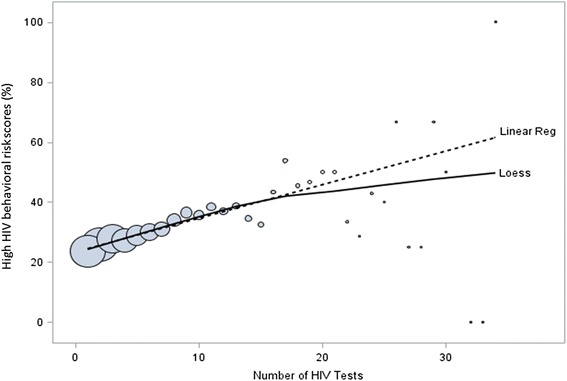
Percentage of high (i.e. ≥5 Menza-score points) HIV behavioral risk scores as modified from [23], by number of HIV tests in repeat testers. The bubble size reflects the denominator used to calculate the percentage of high scores. Linear Reg stands for linear regression, assuming a linear relationship between the y-axis (high risk score) and the x-axis (number of tests). Loess stands for local regression, a smoothing technique that allows one to see a pattern without assuming a particular distribution

To investigate if there was an independent correlation between number of tests over time and change in risk behavior, we performed analyses using the Menza score at every single test as the outcome [23]. All tests with complete data on the variables of the Menza score (10,114 “Early tests” in 2,457 repeat testers) were included. In these 2,457 repeat testers, there was a significant increase of the score between baseline (median, 0; IQR, 0–3) and final test (median, 3; IQR, 0–5; *P* <0.001). The relationship between the number of tests and the logit of high scores (i.e. ≥5; at 28 % of visits subjects reported scores above this cutoff) demonstrates that there was a linear increase of the risk score with log number of tests up to the 17th test (Fig. 2). Univariable mixed-effects logistic regression models (Table 4) showed that more HIV tests (OR = 1.16 for each doubling of the number of tests, *P* <0.001), longer time since first HIV test (OR = 1.08 per year, *P* <0.001), and younger age (OR = 0.96 per 5 year increase of age, *P* = 0.048) were all individually associated with high risk scores (i.e. ≥5). In the multivariable mixed effects model, number of tests (OR = 1.18 for each log_2_ of the number of tests, *P* <0.001) and age (OR = 0.95 per 5-year increase, *P* = 0.006) had significant associations with high risk (i.e. ≥5 points), while time was no longer significant (time and number of tests were highly correlated (r = 0.67)).

**Table 4:** Mixed effects model on factors associated with high-risk behavior: (i.e. ≥5 Menza-score points)

Variables included in the mixed effects model			Univariable		Multivariable	
	N	Number of visits	OR (95 % CI)	*P* value	OR (95 % CI)	*P* value
Number of HIV tests (log_2_)	2457	10,114	1.16 (1.10–1.23)	<0.0001	1.18 (1.11–1.24)	<0.0001
Years since first test	2457	10,114	1.08 (1.04–1.13)	<0.0001	–	n.s.
Age (5-year increase)	2457	10,114	0.96 (0.93–1.00)	0.0479	0.95 (0.92–0.99)	0.0058

A significant association between the number of tests and high risk behavior was demonstrated also for risk behaviors not included in the Menza score, i.e. the combination of CRAI and 10 or more male partners, CRAI with a PWID, and non-injection stimulant drug use (Table 5).

**Table 5:** Associations of number of HIV tests with other risk factors: calculated by univariate mixed effects model by log_2_ increase of tests (i.e. between first, second, fourth, eight, etc.)

Risk factor	Number of individuals	Number of visits	OR for risk factor per log_2_ increase of number of tests		
			OR	(95 % CI)	*P* value
CRAI	2457	10,185	0.99	(0.94–1.04)	n.s.
CRAI and 10 or more male partners	2457	10,223	1.11	(1.05–1.18)	<0.001
CRAI with PWID	2455	10,098	1.35	(1.12–1.64)	0.002
CRAI with sex worker	2456	10,093	1.20	(0.88–1.64)	n.s.
Non-injection stimulant drug use (i.e. methamphetamine, cocaine, poppers, GHB, ketamine, XTC)	2457	10,251	1.37	(1.30–1.45)	<0.001
IDU	2457	10,251	0.91	(0.74–1.13)	n.s.
IDU with Shared needles	2455	10,209	0.95	(0.60–1.50)	n.s.

CRAI, Condomless receptive anal intercourse; GHB, Gamma hydroxybutyrate; IDU, Injection drug use; n.s., Not significant; OR, Odds ratio; PWID, Person who injects drugs; XTC, Ecstasy

## Discussion

This is the largest modern study investigating the relationship between HIV risk behavior and HIV testing among MSM in the United States. Analysis of risk behavior in subjects participating in an AEH screening program (“Early Test”) over the past 6 years identified two major findings. First, MSM who screened repeatedly for HIV reported higher sexual risk behavior at baseline than MSM who only screened once. Second, repeat HIV testing was associated with an increase of risk behavior.

This is the first study to compare baseline risk behavior in single versus repeat testers in an era where serologic test results are available immediately and AEH can be diagnosed by NAT screening. Prior to the era of NAT screening, a handful of studies evaluated risk behaviors in repeat and single testers. In a study by Fernyak [8], the most frequent testers from 1995 to 1997 were those who practiced the highest risk behaviors, and demonstrated the highest incidence of HIV. In another study by MacKellar [24], repeat testers from 1994 to 1998 were more likely to report recent sexual risk behavior and acquire HIV when compared to first-time testers. We found that MSM in San Diego who repeatedly screened for HIV with combined serologic and NAT, practice higher sexual risk behavior at baseline than single testers, but there were no differences identified between these groups for most recreational drugs or recent STI.

This represents the biggest study to date analyzing the associations between repeat testing and risk behavior in repeat testers. In 2007, repeat testers accounted for more than 80 % of HIV tests performed annually in the United States [1], and when analyzing our program since 2008, repeat tests accounted for 48 % (8,396/17,333) of voluntary HIV tests among MSM. Our main finding was that repeat acute infection screening (i.e. rapid serologic testing plus NAT) was independently associated with an increase of risk behaviors between the baseline and the last test. Rates of 10 or more male partners, CRAI with an HIV-positive partner, CRAI with a PWID, non-injection stimulant drug use, and self-reported STIs all increased in line with testing frequency.

To assess change in risk over time we selected a previously validated score for prediction of HIV acquisition among MSM repeat testers as the outcome [23]. When evaluating that Menza risk behavior score at every single “Early test” visit, there was an increase of the proportion of individuals with high scores (i.e. ≥5 points) and the number of tests observed. In the study by Menza, this 5-point cut-off was associated with a cumulative 1-year HIV seroconversion incidence of 3.6 % (derivation cohort) and 4.4 % (validation cohort) [23]. This increase of risk behavior witnessed in our study is unlikely to simply reflect the nation-wide increases in risk behaviors in MSM over time [25], as number of tests – in contrast to time between first and last test – remained an independent predictor of increased risk-behavior in the mixed effects model. This finding may support a hypothesis that provision of negative AEH screening results, which provide HIV status information related to very recent risk behaviors, is independently associated with an increase of risk behaviors between the baseline and the last test. In other words, when a high-risk individual receives a negative HIV result, they may rationalize prior risk behavior as relatively safe, potentially justifying an escalation in subsequent risk [25]. However, although we describe a strong relationship between testing and risk behavior, we are not able to conclude causality, and also the opposite may be true (i.e. clients increased risk behavior is the cause and not the result of repeat testing). Nevertheless, our study provides provocative data that warrants further testing of causation in future studies.

Our study has several additional limitations including its single-center and retrospective design. Further, though pre-test counseling offering personalized risk reduction options was provided at every single “Early Test”, it is likely that the intensity of the counseling may have been reduced at some of the repeated visits. We hypothesized that the frequency of repeat testing is associated with increased risk behavior, but we cannot rule out that repeated tests are not a cause but just a result of increased risk behavior (i.e. our cohort increased their HIV testing as a response to their increased risk behavior). Additionally, testing in primary care settings and even home testing has become more normative during the study period and individuals may have tested outside the “Early Test” or associated studies, which may have led to an underestimation of the proportion of repeat testers. Further, some slight modifications of the previously validated Menza risk score were necessary to fit our available data and analyses. Finally, we cannot completely rule out that subjects reported risk behaviors more honestly with time and repeat testing.

## Conclusions

In conclusion, this study demonstrates that the highest risk individuals for acquiring HIV (e.g. candidates for antiretroviral pre-exposure prophylaxis, referral for additional behavioral counseling, STI screening) that are usually identified by risk scores [26], may also be identified by their testing patterns. Strikingly, we found that repeat HIV testing itself might be associated with an increase in risk behavior. Although we were not able to conclude causality, this finding may support a hypothesis that rapid or immediate feedback of negative test results may reinforce high-risk behavior. To compensate for this reinforcement, intensified pre-test counseling among repeat testers may be necessary, particularly if increasing risk behavior is documented [27]. This might be true especially in the setting of acute infection screening, which provides HIV status information related to very recent risk behaviors. Future studies that assess different intensities and/or types of pre-test counseling (e.g. controlled trial randomizing intervention in high-risk repeat testers to reduce risk-taking behaviors) are needed to assess causality. Such information will be crucial to better understand the dynamics of risk-taking behavior and frame HIV test counseling messages. If true, then new prevention efforts should be developed and evaluated for MSM who repeatedly screen for HIV.

## Abbreviations

AEH: Acute and early HIV infection
AUC: Area under the curve
CI: Confidence interval
CRAI: Condomless receptive anal intercourse
IQR: Inter-quartile range
MSM: Men who have sex with men
n.s.: not significant
NAT: Nucleic acid amplification testing
OR: Odds ratio
PWID: Person who injects drugs
ROC: Receiver operating characteristics
STI: Sexually transmitted infections
